# Pomalidomide and dexamethasone combination with additional cyclophosphamide in relapsed/refractory multiple myeloma (AMN001)—a trial by the Asian Myeloma Network

**DOI:** 10.1038/s41408-019-0245-1

**Published:** 2019-10-08

**Authors:** Cinnie Yentia Soekojo, Kihyun Kim, Shang-Yi Huang, Chor-Sang Chim, Naoki Takezako, Hideki Asaoku, Hideo Kimura, Hiroshi Kosugi, Junichi Sakamoto, Sathish Kumar Gopalakrishnan, Chandramouli Nagarajan, Yuan Wei, Rajesh Moorakonda, Shu Ling Lee, Je Jung Lee, Sung-Soo Yoon, Jin Seok Kim, Chang Ki Min, Jae-Hoon Lee, Brian Durie, Wee Joo Chng

**Affiliations:** 1 National University Cancer Institute, Singapore, Singapore; 2 Sungkyunkwan University, Samsung Medical Center, Seoul, Korea; 3 National Taiwan University, Taipei City, Taiwan; 4 University of Hong Kong, Pok Fu Lam, Hong Kong; 5 National Hospital Organization Disaster Medical Center, Tachikawa, Japan; 6 Hiroshima Red Cross Hospital & Atomic-bomb Survivors Hospital, Hiroshima, Japan; 7 Northern Fukushima Medical Center, Fukushima, Japan; 8 Ogaki Municipal Hospital, Ogaki, Japan; 9 Tokai Central Hospital, Kakamigahara, Japan; 10 Singapore General Hospital, Singapore, Singapore; 11 Singapore Clinical Research Institute, Singapore, Singapore; 12 Cheonnam University Hwasoon Hospital, Hwasun-gun, Korea; 13 Seoul National University Hospital, Seoul, Korea; 14 Yonsei University College of Medicine, Severance Hospital, Seoul, Korea; 15 Seoul St Mary’s Hospital, Seoul, Korea; 16 Gachon University Gil Medical Center, Seongnam-si, Korea; 17 Cedars-Sinai Medical Center, Los Angeles, CA USA

**Keywords:** Myeloma, Phase II trials

## Abstract

Pomalidomide is a third generation immunomodulatory drug which in combination with dexamethasone, has been shown to be active in relapsed/refractory multiple myeloma. However, the data in Asian patients remain limited. We conducted a prospective phase two clinical trial in major cancer centers in Singapore, South Korea, Taiwan, Japan and Hong Kong to assess the efficacy and safety of pomalidomide and dexamethasone combination (PomDex) +/− cyclophosphamide in Asian patients with relapsed/refractory multiple myeloma who failed lenalidomide and bortezomib. Patients were treated with pomalidomide (4 mg daily for 21 days every 4 weeks) and dexamethasone (40 mg weekly). If there is less than a minimal response after three cycles of PomDex, cyclophosphamide 300 mg/m^2^ can be added (PomCyDex). A total of 136 patients were enrolled. The median PFS was 9 and 10.8 months for the PomDex and PomCyDex group, respectively. The median OS was 16.3 months. This regimen appears to be active across age groups and prior lines of treatment. This combination was overall well tolerated with grade 3 and 4 adverse events of mainly cytopenias. PomDex is highly active and well-tolerated in Asian patients. The addition of cyclophosphamide can improve the response and outcomes further in patients with suboptimal response to PomDex.

## Introduction

Multiple myeloma (MM) is a hematological malignancy characterized by clonal expansion of plasma cells in the bone marrow^1^. Advances in treatment including the introduction of proteasome inhibitors and immunomodulatory drugs, such as bortezomib and lenalidomide have significantly improved the survival of MM patients in the last decade^2^ and in the recent years, bortezomib and lenalidomide have been widely used as first or second line therapy in Asia.

Pomalidomide is a third generation immunomodulatory drug that has been shown to have more anti-myeloma effect than thalidomide or lenalidomide^3^. It has limited single agent activity^4,5^ but has been shown to have synergistic effect when combined with dexamethasone^6^. Pomalidomide was approved by FDA in 2013 based on the results of the clinical trial MM-002 study, a multicenter, randomized, open-label study in 221 patients with relapsed/refractory MM who had previously received lenalidomide and bortezomib. In this study, pomalidomide and dexamethasone combination showed superior clinical efficacy with median progression-free survival (PFS) of 4.2 vs. 2.7 months in the pomalidomide only group (hazard ratio = 0.68, *p* = 0.003)^6^. These findings were further confirmed by the MM-003 study, a randomized, open-label, phase three trial comparing pomalidomide plus low-dose dexamethasone vs. high-dose dexamethasone alone in patients with prior exposure to bortezomib and lenalidomide^7^. This study showed that the use of pomalidomide and low-dose dexamethasone improved the median PFS of these patients compared to high-dose dexamethasone alone (4.0 months vs. 1.9 months; hazard ratio = 0·48; *p* < 0·0001)^7^.

In a small randomized Phase 2 study, Baz et al. also showed that the addition of weekly cyclophosphamide to pomalidomide and dexamethasone resulted in improved overall response rates (ORR) compared to pomalidomide and dexamethasone alone (ORR 64.7%; 95% confidence interval [CI] [48.6–80.8%] vs 38.9%; 95% CI [23–54.8%]) (*p* = 0.035); median PFS 9.5 (95% CI, 4.6–14) vs 4.4 (95% CI, 2.3–5.7) months^8^, hence offering a novel option for patients with relapsed/refractory MM.

While the data supporting the efficacy of pomalidomide combinations for the treatment of relapsed/refractory MM has been compelling, most of these studies have been done in the Western population, with Asian subjects accounting for only a small proportion of the total populations enrolled in these clinical studies. Pomalidomide is relatively new in Asia and there remains a need for studies conducted specifically in Asian patients to confirm the efficacy and safety of these combinations in this specific ethnic population. This is particularly of interest in view of the previous report that Asian patients tolerated immunomodulatory drug differently as compared with the Western population, with a higher rate of hematologic toxicity and a lower rate of thromboembolism^9^.

We undertook a prospective phase 2 study to assess the efficacy and safety of pomalidomide and dexamethasone combination (PomDex) in Asian patients with relapsed and/or refractory MM who had failed lenalidomide and relapsed from previous treatment with bortezomib. Our study also aimed to evaluate the efficacy of the addition of weekly cyclophosphamide to pomalidomide and dexamethasone combination (PomCyDex) for patients who had minimal response (MR) or progressive disease with PomDex. The addition of cyclophosphamide is of particular interest as cyclophosphamide is commonly used and readily available in most Asian centers. The possibility of an all oral combination makes this combination particularly attractive. As the doublet of PomDex is well tolerated and efficacious, we would like to see if we could salvage suboptimal responders with the addition of cyclophosphamide, using a response-adapted intensification approach.

## Methods

### Study design and participants

The AMN001 trial is a prospective phase 2 study by the Asian Myeloma Network (AMN) in major cancer centers in Singapore, South Korea, Taiwan, Japan and Hong Kong (NCT02158702).

A total of 100 patients was planned to be recruited into the study based on practical factor as drug support would be provided by Celgene, supplier of pomalidomide for 100 patients. This sample size would provide an adequate cohort to describe the response and toxicity in relation to pomalidomide and dexamethasone in the treatment of MM that relapsed or was refractory to lenalidomide and bortezomib.

For inclusion criteria, patients had to be diagnosed to have refractory or relapsed MM, failed lenalidomide (either refractory to lenalidomide; or no better than stable disease after three cycles of lenalidomide) and relapsed from previous treatment with bortezomib. Patients had to have evaluable disease with serum M-protein ≥ 0.5 g/dL, or in patients without detectable serum M-protein, urine M-protein ≥ 200 mg/24 h, or serum free light chain (sFLC) > 100 mg/L (involved light chain) and an abnormal kappa/lambda ratio. Patients might receive up to six lines of prior treatment. Patients must have Eastern Cooperative Oncology Group (ECOG) Performance Status from 0 to 2. Patients must meet the following clinical laboratory criteria within 21 days of starting treatment: absolute neutrophil count (ANC) ≥ 1000/mm^3^ and platelet ≥ 50,000/mm^3^ (≥ 30,000/mm^3^ if myeloma involvement in the bone marrow is >50%), total bilirubin ≤ 1.5 × the upper limit of the normal range (ULN), alanine aminotransferase (ALT) and aspartate aminotransferase (AST) ≤ 3 × ULN, calculated creatinine clearance ≥ 45 mL/min or creatinine < 3 mg/dL.

The study excluded female patients who were lactating or pregnant, MM of IgM subtype, POEMS syndrome, plasma cell leukemia or circulating plasma cells ≥ 2 × 10^9^/L, Waldenstrom’s Macroglobulinemia and patients with known amyloidosis. Patients could not receive any chemotherapy with approved or investigational anticancer therapeutics within 21 days prior to starting pomalidomide treatment, focal radiation therapy within 7 days prior to start of pomalidomide or radiation therapy to an extended field involving a significant volume of bone marrow within 21 days prior to the start of pomalidomide. Glucocorticoid therapy (prednisolone > 30 mg/day or equivalent) was not allowed within 14 days prior to informed consent obtained. Patients were also not allowed to have immunotherapy (excluding steroids) 21 days prior to the start of pomalidomide. Major surgeries (excluding kyphoplasty) were not allowed within 28 days prior to the start of pomalidomide. This study also excluded active congestive heart failure (New York Heart Association [NYHA] Class III or IV), symptomatic ischemia, or conduction abnormalities uncontrolled by conventional intervention or myocardial infarction within 4 months prior to informed consent obtained. Patients with known HIV seropositive, hepatitis C infection, and/or hepatitis B (except for patients with hepatitis B surface antigen or core antibody receiving and responding to antiviral therapy directed at hepatitis B) were excluded. In addition, the study excluded patients with known cirrhosis, second malignancy within the past 3 years (except for adequately treated basal cell or squamous cell skin cancer, carcinoma in situ of the cervix, breast carcinoma in situ with full surgical resection), myelodysplastic syndrome, and ongoing graft-versus-host disease. Patients with pleural effusions requiring thoracentesis or ascites requiring paracentesis within 14 days prior to starting pomalidomide treatment were excluded. Contraindication to any of the required concomitant drugs or supportive treatments, steroid or lenalidomide hypersensitivity, prior treatment with pomalidomide, clinically significant medical disease or psychiatric condition that in the investigator’s opinion, may interfere with protocol adherence or a patient’s ability to give informed consent were also part of the exclusion criteria.

As pomalidomide is teratogenic, detailed pregnancy avoidance and mitigation strategies were adhered to.

All patients provided written informed consent in accordance with the federal, local and institutional guidelines. The study protocol was approved by the respective appoint Institution Ethics Review Board of the participating centers.

### Drug administration

Patients were treated with oral pomalidomide 4 mg from D1-21 and oral or intravenous dexamethasone 40 mg on days 1, 8, 15, and 22 in a 28-day cycle. Dexamethasone dose was reduced to 20 mg once a week in all patients older than 75 years old. Oral or intravenous cyclophosphamide 300 mg/m^2^ on days 1, 8, and 15 could be added to induce added response if there was a less than MR after three cycles in the absence of disease progression, or if there was disease progression within the first three cycles of pomalidomide and dexamethasone treatment. The options for oral or intravenous cyclophosphamide and dexamethasone were provided to allow physicians to decide based on their institutional guidelines for this multicenter study.

Pomalidomide dose was withheld for grade 3 or 4 non-hematological toxicities or grade 4 hematological toxicity until resolution of these toxicities. On day 1 of the next cycle, the dose of pomalidomide was reduced by 1 mg. Dose levels of pomalidomide were 3 mg (−1 level), and 2 mg (−2 level). Pomalidomide was permanently discontinued in the event of grade 4 rash or rash with blistering, or grade 4 peripheral neuropathy.

Dose modifications for dexamethasone and cyclophosphamide were at the discretion of the investigators. The dose levels for dexamethasone were 20 mg (−1 level), 12 mg (−2 level) and 8 mg (−3 level). The dose levels for cyclophosphamide were 200 mg/m^2^ (−1 level), and 100 mg/m^2^ (−2 level).

Concomitant use of bisphosphonate was allowed. Thromboprophylaxis with low-dose aspirin (100 mg or less) was allowed although not mandated, based on the previous study which showed low rate of venous thromboembolism (VTE) in Asian patients treated with thalidomide and thromboprophylaxis with aspirin or warfarin did not reduce risk of VTE^10^. Prophylaxis against Pneumocystis carinii (PCP) with Bactrim and Herpes with acyclovir and the use of proton pump inhibitor was allowed according to institutional practice. Allopurinol to prevent tumor lysis was allowed at the discretion of the treating physician. Focal radiotherapy to lytic lesion for pain control was allowed.

### Primary and secondary endpoints

The primary endpoint was PFS, defined as the time from commencement of treatment with pomalidomide and dexamethasone to disease progression or death due to any cause, whichever occurred first.

The secondary endpoints were ORR, defined as the percentage of patients enrolled that achieved a complete response (CR), or stringent complete response (sCR), or very good partial response (VGPR), or partial response (PR) based on the International Myeloma Working Group criteria^11^ anytime from the commencement of treatment with pomalidomide and dexamethasone to the end of study and duration of response (DOR), defined as the time from first evidence of PR or better to confirmation of disease progression or death due to any cause. All response categories (CR, sCR, VGPR, PR) and disease progression required two consecutive assessments. Other secondary endpoints include overall survival (OS) defined as the time from commencement of treatment with pomalidomide and dexamethasone to death due to any cause.

Adverse events were graded according to the National Cancer Institute’s Common Terminology Criteria for Adverse Events version 4.03. Safety assessment was based on reported adverse events, clinical laboratory tests, vital signs, physical examinations, and ECOG performance status.

### Statistical analysis

Continuous variables were summarized using descriptive statistics, i.e., mean, standard deviation, median, minimum, and maximum. Categorical variables were summarized by frequencies and percentages. The duration of PFS was censored for patients who meet 1 of the following conditions: (1) Starting new anticancer therapy before documentation of disease progression or death; (2) Death or disease progression immediately after more than 1 consecutively missed disease assessment visit or; (3) Alive without documentation of disease progression before end of the study. The DOR was censored based on the same censoring conventions defined for PFS. The duration of OS was censored for patients who were alive or lost to follow-up at the patient’s date of last contact (last known to be alive). Kaplan–Meier method was used to estimate median PFS, OS and DOR and 95% CI were calculated using the Brookmeyer and Crowley method. Cox regression was used to test the difference in PFS between the following subgroups: (1) 1–3 prior lines of treatment vs. more than 3 prior lines of treatment; (2) 65 years old and below vs. above 65 years old; (3) Highest response of PR and above vs. stable and MR vs. disease progression; (4) ISS 1–2 vs. ISS 3; 5) HR genetics (t(4;14), 17p13del) vs. others. Hazard ratios and 95% CIs were reported. A *p*-value < 0.05 was considered statistically significant. All statistical analyses were carried out using SAS (Statistical Analysis System software, SAS Institute, North Carolina, USA) version 9.4. Subgroup analyses were performed in patients who received cyclophosphamide as well as in patients who had prior treatment with novel agents such as carfilzomib, ixazomib, panobinostat, elotuzumab or daratumumab.

## Results

### Patient characteristics

**Fig. 1:**
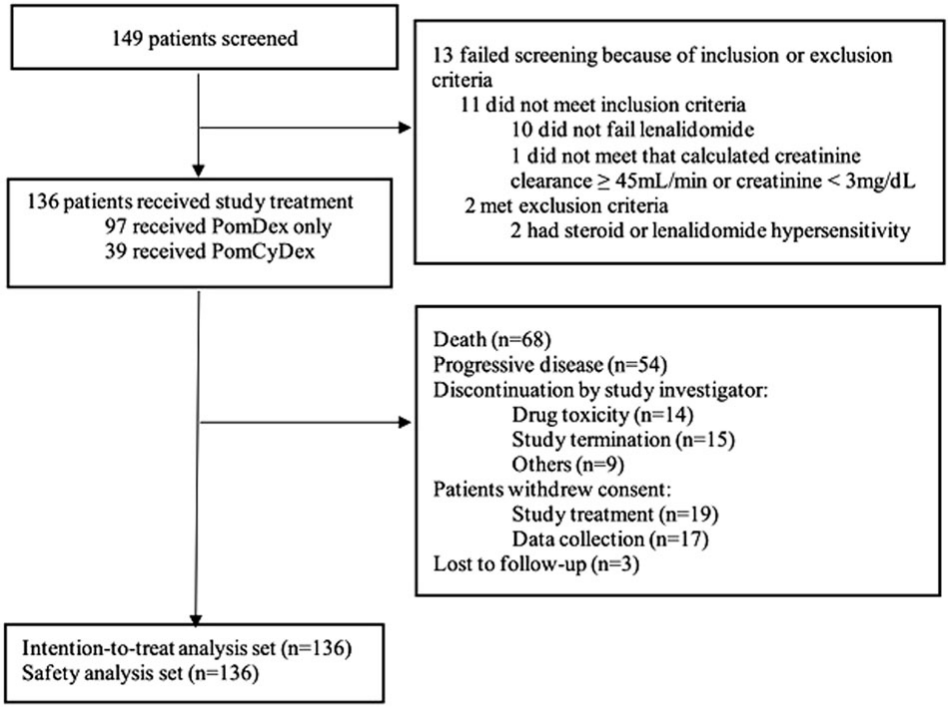
CONSORT flow diagram ▓

Between December 2014 to February 2017, a total of 136 patients were enrolled (Fig. 1). Baseline characteristics are listed on Table 1. Median age was 66 years. 90.4% of patients had ECOG performance status of 0 or 1. International Staging System (ISS) were I (37.5%), II (33.8%), or III (25.7%) with 3% missing data. Of 44 patients who had fluorescence in situ hybridization (FISH) done, 61.4% had high-risk FISH abnormalities as described in the R-ISS staging system^12^.

**Table 1:** Baseline patient characteristics and FISH test characteristics

Demographic Parameter (*N* = 136)	*N* (%)
*Gender*	
Male	72 (52.9)
Female	64 (47.1)
*Age (years)*	
=<65	66 (48.5)
>65	70 (51.5)
*ECOG Performance score*	
Grade 0	63 (46.3)
Grade 1	60 (44.1)
Grade 2	13 (9.6)
*International staging system*	
Stage I	51 (37.5)
Stage II	46 (33.8)
Stage III	35 (25.7)
Missing	4 (2.9)
*Previous treatments*	
Autologous transplant	68 (50)
Bortezomib	135 (99.3)
Thalidomide	85 (62.5)
Lenalidomide	136 (100)

FISH Tests (*N* = 44)	*N* (%)
*t(4;14)*	
Positive	15 (34.1)
Negative	24 (54.5)
Not done	5 (11.4)
*t(11;14)*	
Positive	3 (6.8)
Negative	22 (50.0)
Not done	19 (43.2)
*t(14;16)*	
Positive	4 (9.1)
Negative	26 (59.1)
Not done	14 (31.8)
*17p13 Deletion*	
Positive	8 (18.2)
Negative	27 (61.4)
Not done	9 (20.5)
*13 Deletion*	
Positive	17 (38.6)
Negative	11 (25)
Not done	16 (36.4)
*1q21 Amplification*	
Positive	8 (18.2)
Negative	8 (18.2)
Not done	28 (63.6)

Data are number (%)

*ECOG* Eastern Cooperative Oncology Group

Except for one patient who was not previously treated with bortezomib, the rest of the patients had previous bortezomib and lenalidomide treatment, while 18.4% had also received other novel agents such as carfilzomib, ixazomib, panobinostat, elotuzumab or daratumumab prior to study enrollment. A total of 68 (50%) patients had prior autologous stem cell transplantation. There were 70 (51.5%) patients who had 1–3 prior treatment lines and 66 (48.5%) patients who had more than 3 prior treatment lines. The median number of cycles was 7 cycles. The median duration of follow-up was 358.5 days.

### Efficacy

Of the 136 patients, 97 patients received pomalidomide and dexamethasone (PomDex) and 39 patients received additional cyclophosphamide (PomCyDex).

**Fig. 2:**
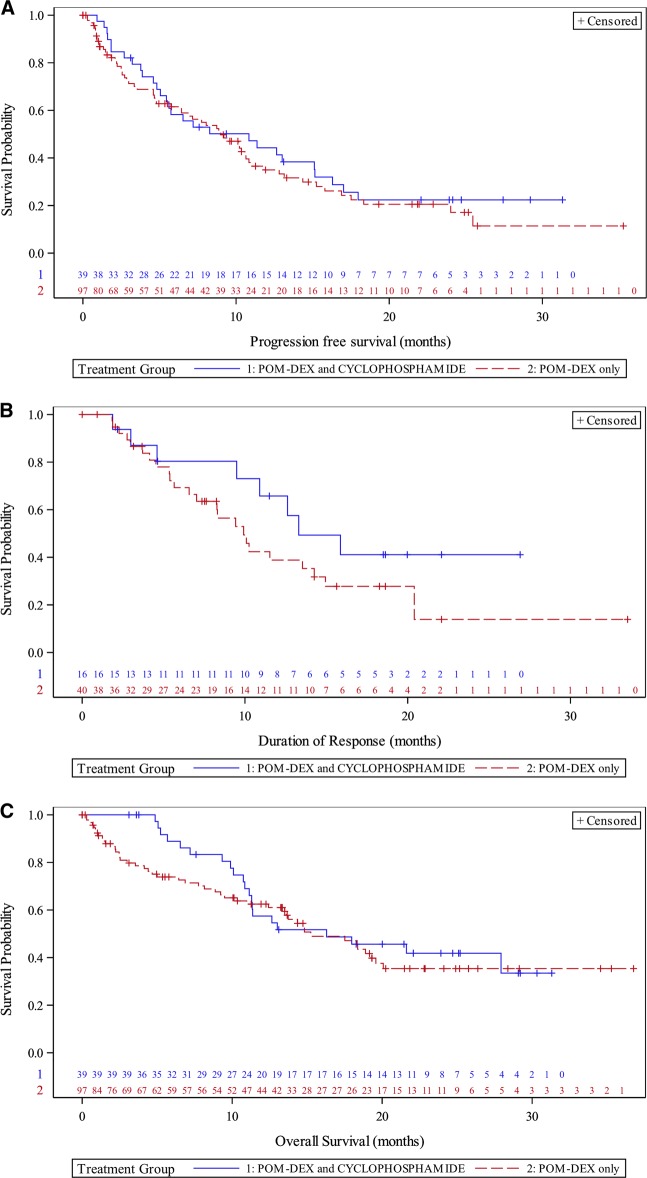
**a** Progression-free survival (PFS) in patients treated with PomDex and PomCyDex. Kaplan–Meier curves and median PFS in patients on PomDex and PomCyDex **b** Duration of response (DOR) in patients treated with PomDex and PomCyDex. Kaplan–Meier curves and median DOR in patients on PomDex and PomCyDex. **c** Overall survival (OS) in patients treated with PomDex and PomCyDex. Kaplan–Meier curves and median OS in patients on PomDex and PomCyDex

The overall median PFS (*N* = 136) was 9 months (95% CI, 6.44–10.84). The median PFS for the PomDex group was 9 months (95% CI, 6.44–10.84). Interestingly, patients who had less than MR or disease progression for whom cyclophosphamide was added (PomCyDex group), had longer median PFS of 10.8 months (95% CI, 5.06–15.15) (Fig. 2a).

Of 110 evaluable patients, the ORR was 51.8%. The ORR was 56.3% (40 out of 71 patients) in the PomDex group, including CR or sCR in 4 patients (5.6%), VGPR in 10 patients (14.1%), and PR in 26 patients (36.6%). For the PomCyDex group, the ORR was 43.6% (17 out of 39 patients), including CR or sCR in 1 patient (2.6%), VGPR in 3 patients (7.7%), and PR in 13 patients (33.3%) (Table 2). The median DOR was 12.6 months (95%CI, 9.43–15.87). The median DOR was 10.1 months (95% CI, 8.28–15.05) in the PomDex group and 15.9 months (95%CI, 9.49-not estimable) in the PomCyDex group (Fig. 2b).

**Table 2:** Response rate

	All (*N* = 110) *N* (%)	PomDex Only (*N* = 71) *N* (%)	PomCyDex (*N* = 39) *N* (%)
Overall response	57 (51.8)	40 (56.3)	17 (43.6)
CR or sCR	5 (4.5)	4 (5.6)	1 (2.6)
VGPR	13 (11.8)	10 (14.1)	3 (7.7)
PR	39 (35.5)	26 (36.6)	13 (33.3)
MR	11 (10.0)	6 (8.5)	5 (12.8)
SD	29 (26.4)	18 (25.4)	11 (28.2)
PD	13 (11.8)	7 (9.9)	6 (15.4)

Data are number (%)

*CR*, complete response, *sCR* stringent complete response, *VGPR* very good partial response, *PR* partial response, *MR* minimal response, *SD* stable disease, *PD* progressive disease

The median overall survival (OS) was 16.3 months (95% CI, 12.65–20.07). The PomDex group had median OS of 15.2 months (95% CI, 12.42–19.58). Similar to the PFS result above, patients who had less than MR or disease progression for whom cyclophosphamide was added, had longer median OS of 16.3 months (PomCyDex group) (95% CI, 11.14-not estimable) (Fig. 2c).

**Fig. 3:**
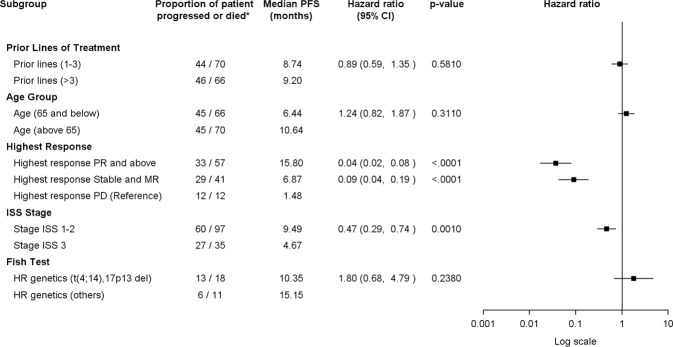
Forest plot for progression free survival by patients’ characteristics. Forest plot, proportion of patient progressed or died, median PFS, hazard ratio and *p*-value by patients’ characteristics

On subgroup analysis, median PFS was significantly better in the groups that achieved deeper response, with median PFS of 15.8 months (HR 0.04, 95% CI[0.02–0.08]) for those with best response of PR or better, 6.87 months (HR 0.09, 95% CI [0.04–0.19]) for those with best response of stable disease (SD) and MR, as compared to 1.6 months for those with disease progression (*p* < 0.0001). Patients with ISS stages 1–2 had better median PFS of 9.5 months (HR 0.47, 95% CI [0.29–0.74]) as compared to the patients with ISS stage 3 with median PFS of 4.7 months (*p* = 0.0010). We found that pomalidomide is active in patients with high-risk genetics with a median PFS of 10.4 months in the patients with 17pdel or t(4;14) and 15.2 months in the patients with other genetic abnormalities. (Fig. 3).

There was no observed difference in PFS by age (65 years old and below vs more than 65 years old) and number of prior lines of treatment (1–3 vs. more than 3 lines).

From 20 evaluable patients with prior treatment with novel agents, such as carfilzomib, ixazomib, panobinostat, elotuzumab or daratumumab, 53.8% of patients in the PomDex group (7 out of 13) showed response, including 2 patients who achieved VGPR and 5 patients who achieved PR. In the PomCyDex group (*N* = 7), 2 patients achieved PR and no patients achieved more than PR (Table 3). The median DOR in this cohort (with at least PR) was 7.23 months (95% CI, 1.84–23.20) in the PomDex group and 8.95 months (95% CI, 4.60–13.31) in the PomCyDex group.

**Table 3:** Subgroup analysis of response rate: patients with prior treatment of carfilzomib, ixazomib, panobinostat, elotuzumab or daratumumab

	All (*N* = 20) *N* (%)	PomDex only (*N* = 13) *N* (%)	PomCyDex (*N* = 7) *N* (%)
VGPR	2 (10.0)	2 (15.4)	0 (0.0)
PR	7 (35.0)	5 (38.5)	2 (28.6)
MR	2 (10.0)	1 (7.7)	1 (14.3)
SD	4 (20.0)	4 (30.8)	0 (0.0)
PD	5 (25.0)	1 (7.7)	4 (57.1)

Data are number (%)

*VGPR* very good partial response, *PR* partial response, *MR* minimal response, *SD* stable disease, PD progressive disease

### Safety and tolerability

The treatment was overall well tolerated. The adverse events are listed in Table 4. Grade 3 and 4 adverse events were mainly cytopenias including anemia (18.4% patients, 43 events), neutropenia (49.3% patients, 121 events), and thrombocytopenia (19.1% patients, 51 events). Overall, 42.4% of the anemia, 81.6% of the neutropenia, and 33.3% of the thrombocytopenia events were deemed to be related to study treatment.

**Table 4:** Adverse events

Summary (frequency and percentages) of number of episodes of AEs							
Adverse events (AEs), *n* (%) [nAE]	Toxicity Grade					Relationship to study treatment	
	Grade 1	Grade 2	Grade 3	Grade 4	Grade 5	Related	Not related
Total	76 (55.9) [353]	82 (60.3) [461]	95 (69.9) [364]	34 (25.0) [83]	36 (26.5) [39]	97 (71.3) [478]	102 (75.0) [822]
Abnormal liver function test	2 (1.5) [4]	3 (2.2) [4]	1 (0.7) [1]	0	0	2 (1.5) [2]	3 (2.2) [7]
Anemia	5 (3.7) [6]	14 (10.3) [17]	23 (16.9) [38]	2 (1.5) [5]	0	15 (11.0) [28]	19 (14.0) [38]
Anorexia	8 (5.9) [9]	8 (5.9) [9]	2 (1.5) [2]	0	0	9 (6.6) [9]	10 (7.4) [11]
Anxiety	1 (0.7) [1]	1 (0.7) [1]	0	0	0	0	2 (1.5) [2]
Blurred vision	2 (1.5) [2]	0	0	0	0	1 (0.7) [1]	1 (0.7) [1]
Constipation	17 (12.5) [19]	9 (6.6) [14]	1 (0.7) [1]	0	0	8 (5.9) [8]	17 (12.5) [26]
Cough	12 (8.8) [14]	11 (8.1) [11]	0	0	0	2 (1.5) [2]	21 (15.4) [23]
Diarrhea	6 (4.4) [6]	13 (9.6) [17]	3 (2.2) [4]	1 (0.7) [1]	0	5 (3.7) [7]	15 (11.0) [21]
Dizziness	4 (2.9) [4]	2 (1.5) [2]	0	0	0	3 (2.2) [3]	3 (2.2) [3]
Dyspnea	9 (6.6) [9]	2 (1.5) [2]	5 (3.7) [6]	0	0	6 (4.4) [6]	11 (8.1) [11]
Edema	6 (4.4) [11]	9 (6.6) [13]	4 (2.9) [4]	0	0	11 (8.1) [19]	6 (4.4) [9]
Fatigue	19 (14.0) [24]	7 (5.1) [8]	4 (2.9) [5]	0	0	17 (12.5) [25]	9 (6.6) [12]
Fever	8 (5.9) [17]	12 (8.8) [14]	7 (5.1) [10]	0	0	9 (6.6) [17]	16 (11.8) [24]
Headache	1 (0.7) [2]	0	1 (0.7) [1]	0	0	1 (0.7) [1]	2 (1.5) [2]
Heart Failure	0	0	2 (1.5) [2]	0	2 (1.5) [2]	2 (1.5) [2]	2 (1.5) [2]
Hypercalcaemia	2 (1.5) [2]	3 (2.2) [6]	1 (0.7) [1]	0	0	0	5 (3.7) [9]
Hyperkalemia	4 (2.9) [4]	3 (2.2) [7]	2 (1.5) [2]	0	0	0	7 (5.1) [13]
Hypernatremia	1 (0.7) [1]	0	1 (0.7) [1]	0	0	0	1 (0.7) [2]
Hypertension	2 (1.5) [2]	1 (0.7) [1]	0	0	0	2 (1.5) [2]	1 (0.7) [1]
Hypocalcaemia	5 (3.7) [5]	5 (3.7) [7]	0	0	0	1 (0.7) [1]	8 (5.9) [11]
Hypokalemia	6 (4.4) [6]	4 (2.9) [7]	2 (1.5) [6]	1 (0.7) [1]	0	2 (1.5) [2]	9 (6.6) [18]
Hyponatremia	1 (0.7) [1]	1 (0.7) [1]	2 (1.5) [2]	0	0	1 (0.7) [1]	3 (2.2) [3]
Hypotension	2 (1.5) [2]	5 (3.7) [8]	1 (0.7) [1]	0	0	1 (0.7) [1]	7 (5.1) [10]
Insomnia	5 (3.7) [7]	8 (5.9) [10]	3 (2.2) [4]	0	0	8 (5.9) [13]	6 (4.4) [8]
Muscle spasms	5 (3.7) [6]	2 (1.5) [2]	1 (0.7) [2]	0	0	4 (2.9) [4]	3 (2.2) [6]
Muscle weakness	1 (0.7) [1]	3 (2.2) [3]	0	0	0	0	4 (2.9) [4]
Nausea	4 (2.9) [4]	3 (2.2) [3]	0	0	0	2 (1.5) [2]	5 (3.7) [5]
Neuralgia	0	3 (2.2) [3]	0	0	0	1 (0.7) [1]	2 (1.5) [2]
Neutropenia	1 (0.7) [1]	17 (12.5) [25]	44 (32.4) [89]	23 (16.9) [32]	0	47 (34.6) [120]	14 (10.3) [27]
Neutropenic fever	1 (0.7) [1]	0	12 (8.8) [14]	0	1 (0.7) [1]	10 (7.4) [12]	4 (2.9) [4]
Pain	5 (3.7) [5]	4 (2.9) [9]	2 (1.5) [2]	0	0	1 (0.7) [1]	8 (5.9) [15]
Peripheral neuropathy	7 (5.1) [9]	2 (1.5) [2]	1 (0.7) [2]	0	0	7 (5.1) [10]	3 (2.2) [3]
Pleural effusion	1 (0.7) [1]	0	1 (0.7) [2]	0	1 (0.7) [1]	1 (0.7) [3]	1 (0.7) [1]
Pneumonia	0	6 (4.4) [6]	26 (19.1) [29]	3 (2.2) [3]	7 (5.1) [7]	16 (11.8) [17]	23 (16.9) [28]
Rash	10 (7.4) [11]	5 (3.7) [5]	4 (2.9) [4]	0	0	13 (9.6) [13]	6 (4.4) [7]
Renal impairment	2 (1.5) [2]	2 (1.5) [5]	4 (2.9) [4]	2 (1.5) [2]	0	2 (1.5) [2]	7 (5.1) [11]
Sepsis	1 (0.7) [1]	1 (0.7) [1]	8 (5.9) [9]	4 (2.9) [4]	7 (5.1) [7]	6 (4.4) [6]	12 (8.8) [16]
Thrombocytopenia	7 (5.1) [7]	6 (4.4) [9]	14 (10.3) [28]	12 (8.8) [23]	0	16 (11.8) [23]	11 (8.1) [44]
Upper respiratory tract infection	4 (2.9) [4]	14 (10.3) [24]	2 (1.5) [3]	0	0	3 (2.2) [3]	16 (11.8) [28]
Urinary tract infection	2 (1.5) [2]	4 (2.9) [7]	1 (0.7) [1]	0	0	0	7 (5.1) [10]
Venous thrombosis	1 (0.7) [1]	1 (0.7) [1]	3 (2.2) [3]	0	0	2 (1.5) [4]	1 (0.7) [1]
Vomiting	1 (0.7) [1]	4 (2.9) [7]	0	0	0	3 (2.2) [4]	3 (2.2) [4]
Others, specify	54 (39.7) [138]	51 (37.5) [190]	41 (30.1) [81]	10 (7.4) [12]	21 (15.4) [21]	44 (32.4) [103]	78 (57.4) [339]

Incidence of grade > = 3 sepsis was 13.9%. The incidence of neutropenic fever was 10.2%. One patient died from neutropenic sepsis.

There were 3 patients who had in total 5 thromboembolic events, 2 of the patients were on aspirin prophylaxis while 1 of the patients was not on any prophylaxis. Peripheral neuropathy was seen in 10 patients, mostly grade 1 (7 patients, 9 events) and grade 2 (2 patients, 2 events). One patient had grade 3 with none having grade 4 peripheral neuropathy.

Dose modification was needed for 40.4%, 27.9%, and 28.7% of patients for pomalidomide, dexamethasone, and cyclophosphamide, respectively. Overall drug discontinuation rate was 22.8% (31 out of 136 patients). There were 69 (50.7%) patients who had drug interruption and 30 (22.1%) patients who had dose reduction due to adverse events.

Grade 5 adverse events (resulting in death) happened to 36 patients; 33 (91.7%) were deemed unrelated to the study treatment and 3 (8.3%) of the 36 patients were deemed to have treatment-related deaths (5 events were reported as one patient had pneumonia, heart failure and atrioventricular block, one patient had pleural effusion, and one patient had neutropenic sepsis). There were 15 patients who died from infective complications and 6 patients died from disease progression or refractory MM. Two patients died from other tumors, one with carcinoma of unknown origin and another one with submucosal gastric tumor.

## Discussions

AMN001 study is the first prospective Asian study which evaluated the efficacy and safety of PomDex combination with and without the addition of cyclophosphamide (PomCyDex) in patients with relapsed/refractory MM who had failed lenalidomide and relapsed from previous bortezomib treatment. Patients were treated with PomDex and if there was less than MR after 3 cycles of PomDex, cyclophosphamide 300 mg/m^2^ can be added (PomCyDex).

Our findings confirmed the efficacy of this combination, with a median PFS of 9 months, OS of 16.3 months and ORR of 51.8%. Our findings also showed that the addition of cyclophosphamide to patients who had inadequate response to PomDex was able to salvage the response with 43.6% of these patients achieving PR or better, including 7.7% of patients achieving VGPR and 2.6% of patients achieving CR or sCR, and these patients had even longer median PFS and OS (10.8 months (95% CI, 5.06–15.15) and 16.3 months (95% CI, 11.14-not estimable), respectively).

These efficacy outcomes compared favorably with those previously described in phase 2 or 3 studies in similar patient population^7,8,13–15^. Our results support the previous findings^7^ that pomalidomide was able to induce response in lenalidomide-refractory MM patients. This could be explained by preclinical studies which showed that there were differential mechanisms in the acquired resistance for lenalidomide-dexamethasone and pomalidomide-dexamethasone combination and they did not display cross-resistance^16^.

Of note, the observed median PFS (9 months) was longer as compared to the previous studies (4.2–4.6 months)^7,13^ (Table 5). This median PFS compared favorably with that reported in another phase 2 PomCyDex study by Baz et al. in relapsed/refractory myeloma^8^. The outcome benefit appeared similar regardless of the number of prior lines of therapy used. Better outcomes were seen in patients with ISS stage 1–2 as compared to stage 3.

**Table 5:** Comparison with other studies

Phase (Trial) (Sample size)	P3 (MM-003) (*N* = 455)	P3 (MM-010) (*N* = 676)	P2 (Baz et al.) (*N* = 70) (PomDex: *n* = 36) (PomCyDex: *n* = 34)		P2 (AMN001) (*N* = 136)
	PomDex	PomDex	PomDex	PomCyDex	Pom(Cy)Dex
ORR (%)	31	32.6	38.9	64.7	51.8
PFS (months)	4.0	4.6	4.4	9.5	9.0
OS (months)	12.7	11.9	16.8	NR	16.3

Our results suggested that the addition of cyclophosphamide to the PomDex combination was able to salvage the suboptimal response in the cohort with progressive disease or less than MR to the PomDex combination alone. PomCyDex combination was also able to achieve longer DOR.

As compared with previous small phase 2 Asian study^17^, our ORR is better (51.8% vs. 42%), which might be contributed by the addition of cyclophosphamide for patients with inadequate response in our cohort.

Our results also supported the previous study result^18^ that PomDex is active in MM with high-risk genetics including 17pdel and t(4;14). This is particularly important as patients with high-risk genetics typically do not respond as well to conventional treatment and this combination stands as a good option for these patients. It might be interesting to evaluate the efficacy of this combination in patients with t(4;14) or 17pdel separately in future Asian studies.

Another interesting finding was that in the subgroup analysis of 20 patients with prior treatment of carfilzomib, ixazomib, panobinostat, elotuzumab or daratumumab, response was seen in more than half of the patients (53.8%) with PomDex combination and for the patients who had suboptimal response to the PomDex combination, PomCyDex was able to induce partial response in 2 out of 7 patients. This result shows that PomDex might be a feasible option in the later stage of MM treatment. It might be interesting to evaluate in further studies if this might be an option to bridge to the CAR-T cell therapies or bispecific T-cell engagers.

In our Asian study population, we found that the PomDex and PomCyDex combinations were well tolerated, with adverse event rate similar to the previously reported global studies^13,19,7^ Similar to previous studies, the most common adverse event was myelosuppression. The incidence of > = grade 3 sepsis was lower than the MM-003 study^7^ (30% vs. 13.9%) and similar with previous Asian study (8.3%)^17^. Similar to previous global and Asian studies^7,17,20^ VTE rate was low and peripheral neuropathy was manageable with mostly grade 1–2 events.

One limitation of our study was in its single arm open-label study design with no comparator arm. Nevertheless, we were able to demonstrate clinically significant PFS and ORR benefit in our Asian population.

In conclusion, Pomalidomide and dexamethasone combination is an efficacious and safe option in Asian patients with relapsed/refractory MM who were refractory to lenalidomide and relapsed from previous bortezomib treatment, and the addition of cyclophosphamide in patients with suboptimal response can improve the outcomes further. This regimen appears to be active across age groups and prior lines of treatment. In particular, it is very active even in patients who have progressed following treatment with the latest generations of approved drugs including monoclonal antibodies. The possibility of oral route of administration makes this combination particularly attractive. With the improvement of drug access in the Asian countries in which bortezomib and/or lenalidomide therapies have increasingly become the standard of care in MM management during induction therapy and/or early relapse, this combination could be an appealing treatment option in the relapsed/refractory MM patients.

## References

[1] Kyle RA, Rajkumar SV (2008). Multiple myeloma. Blood..

[2] Kumar SK (2008). Improved survival in multiple myeloma and the impact of novel therapies. Blood..

[3] Quach H (2010). Mechanism of action of immunomodulatory drugs (IMiDS) in multiple myeloma. Leukemia..

[4] Schey SA (2004). Phase I study of an immunomodulatory thalidomide analog, CC-4047, in relapsed or refractory multiple myeloma. J. Clin. Oncol..

[5] Streetly MJ (2008). Alternate day pomalidomide retains anti-myeloma effect with reduced adverse events and evidence of in vivo immunomodulation. Br. J. Haematol..

[6] Richardson PG (2014). Pomalidomide alone or in combination with low-dose dexamethasone in relapsed and refractory multiple myeloma: a randomized phase 2 study. Blood..

[7] Miguel JS (2013). Pomalidomide plus low-dose dexamethasone versus high-dose dexamethasone alone for patients with relapsed and refractory multiple myeloma (MM-003): a randomised, open-label, phase 3 trial. Lancet Oncol..

[8] Baz RC (2016). Randomized multicenter phase 2 study of pomalidomide, cyclophosphamide, and dexamethasone in relapsed refractory myeloma. Blood..

[9] Lu J (2017). Continuous treatment with lenalidomide and low-dose dexamethasone in transplant-ineligible patients with newly diagnosed multiple myeloma in Asia: subanalysis of the FIRST trial. Br. j. Haematol..

[10] Kato A (2013). A retrospective cohort study of venous thromboembolism(VTE) in 1035 Japanese myeloma patients treated with thalidomide; lower incidence without statistically significant association between specific risk factors and development of VTE and effects of thromboprophylaxis with aspirin and warfarin. Thromb. Res..

[11] Kumar S (2016). International Myeloma Working Group consensus criteria for response and minimal residual disease assessment in multiple myeloma. Lancet Oncol..

[12] Palumbo A (2015). Revised International Staging System for Multiple Myeloma: A Report From International Myeloma Working Group. J. Clin. Oncol..

[13] Dimopoulos MA (2016). Safety and efficacy of pomalidomide plus low-dose dexamethasone in STRATUS (MM-010): a phase 3b study in refractory multiple myeloma. Blood..

[14] Leleu X (2013). Pomalidomide plus low-dose dexamethasone is active and well tolerated in bortezomib and lenalidomide-refractory multiple myeloma: Intergroupe Francophone du Myelome 2009-02. Blood..

[15] Ailawadhi S (2018). Pomalidomide-dexamethasone in refractory multiple myeloma: long-term follow-up of a multi-cohort phase II clinical trial. Leukemia..

[16] Ocio EM (2015). In vivo murine model of acquired resistance in myeloma reveals differential mechanisms for lenalidomide and pomalidomide in combination with dexamethasone. Leukemia..

[17] Ichinohe T (2015). A multicenter phase 2 study of pomalidomide plus dexamethasone in patients with relapsed and refractory multiple myeloma: the Japanese MM-011 trial. Exp. Hematol. Oncol..

[18] Leleu X (2015). Pomalidomide plus low-dose dexamethasone in multiple myeloma with deletion 17p and/or translocation (4;14): IFM 2010-02 trial results. Blood..

[19] Garderet L (2018). Pomalidomide, cyclophosphamide, and dexamethasone for relapsed multiple myeloma. Blood..

[20] Matsue K (2015). Pomalidomide alone or in combination with dexamethasone in Japanese patients with refractory or relapsed and refractory multiple myeloma. Cancer Sci..

